# Genome-wide characterization and expression profiling of the *Phospholipase C* (*PLC*) gene family in three orchids of economic importance

**DOI:** 10.1186/s43141-021-00217-z

**Published:** 2021-08-21

**Authors:** Madhvi Kanchan, Thakku R. Ramkumar, Himani, Jaspreet K. Sembi

**Affiliations:** 1 Department of Botany, Panjab University, Chandigarh, 160014 India; 2 Department of Microbiology and Cell Sciences, University of Florida, Gainesville, FL 32611 USA

**Keywords:** Orchids, Phospholipase, *Phalaenopsis equestris*, *Dendrobium catenatum*, *Apostasia shenzhenica*

## Abstract

**Background:**

Phospholipases hydrolyze glycerophospholipids and generate diverse lipid-derived molecules with secondary messenger activity. Out of these, phospholipase C (PLC) specifically cleaves the phospholipids at ester linkages and yields diacylglycerol (DAG) and phosphorylated head groups. PLCs are classified further as phosphatidylinositol-specific PLCs (PI-PLCs) and non-specific PLCs with biased specificity for phosphatidylcholine (NPC/PC-PLC).

**Results:**

In the present report, we identified and characterized *PLC* genes in the genomes of three orchids, *Phalaenopsis equestris* (seven *PePLCs*), *Dendrobium catenatum* (eight *DcPLCs*), and *Apostasia shenzhenica* (seven *AsPLCs*). Multiple sequence alignment analysis confirmed the presence of conserved X and Y catalytic domains, calcium/lipid-binding domain (C2 domain) at the C terminal region, and EF-hand at the N-terminal region in PI-PLC proteins and esterase domain in PC-PLC. Systematic phylogenetic analysis established the relationship of the PLC protein sequences and clustered them into two groups (PI-PLC and PC-PLC) along with those of *Arabidopsis thaliana* and *Oryza sativa*. Gene architecture studies showed the presence of nine exons in all *PI-PLC* genes while the number varied from one to five in *PC-PLCs*. RNA-seq-based spatio-temporal expression profile for *PLC* genes was generated, which showed that *PePC-PLC1*, *PePC-PLC2A*, *DcPC-PLC1A*, *DcPC-PLC1B*, *DcPC-PLC2*, *DcPC-PLC1B*, and *AsPC-PLC1* had significant expression in all reproductive and vegetative tissues. The expression profile is matched to their upstream *cis*-regulatory promoter elements, which indicates that *PLC* genes have a role in various growth and development processes and during stress responses.

**Conclusions:**

The present study unwrapped the opportunity for functional characterization of selected *PLC* genes in planta for plant improvement.

**Supplementary Information:**

The online version contains supplementary material available at 10.1186/s43141-021-00217-z.

## Background

The plasma membrane acts as a barrier between cells and the outside environment and plays a major role in the development and protection of plants from external stresses. Phospholipids act as building blocks of the plasma membrane; the composition of these compounds dynamically change, during various growth and developmental processes and in response to abiotic and biotic stresses [1]. These compositional changes are essential for the maintenance of membrane integrity and stability, which is necessary for the overall health and growth of plants. The phospholipase superfamily is a large family of enzymes, which is involved in the above process. Phospholipases are considered a diverse group of principle enzymes involved in lipid hydrolysis [2]. Essentially, all the lipid derivatives are supposed to play a major role as signaling compounds in various cellular processes. The phospholipase superfamily on the basis of their substrate specificity is categorized into three sub-families, phospholipase A (PLA), phospholipase C (PLC), and phospholipase D (PLD). Phospholipase C is considered one of the essential lipid-hydrolyzing enzymes; it cleaves the ester linkage of phospholipid molecule of the plasma membrane and yields a water-soluble phospholipid head group and diacylglycerol (DAG) [3]. The phospholipase C sub-family is further classified on the basis of the affinity toward the phospholipid head group as the substrate, into two categories, phosphatidylinositol-specific phospholipase C (PI-PLC) and non-specific phospholipase with biased specificity for phosphatidylcholine (PC-PLC/NPC). PC-PLC mainly hydrolyze the membrane lipids (phosphatidylcholine and phosphatidylethanolamine) and acts in a calcium-independent manner [4–8]. In addition to this, it is reported that in plant cells phosphorylated products of DAG, the phosphatidic acid (PA), diacylglycerol pyrophosphate (DGPP), and hexakisphosphate (IP6) acts as secondary messengers [9]. The *PLC* gene family was first reported in *Arabidopsis thaliana*, where nine *PI-PLC* and six *PC-PLC* genes were identified [10, 11]. After *Arabidopsis*, *PLC* genes have been identified from a number of plant species such as *Oryza sativa* [12], *Triticum turgidum* [13], *Gossypium hirsutum*, *G. arboretum*, *and G. raimondii* [9], *Lycopersicon esculentum* [14], *Glycine max* [5, 15], *Solanum tuberosum* [16], *Pisum sativum* [17], *Brassica napus* [18, 19], *Vigna radiata* L. [20], *Avena sativa* [21], *Lilium daviddi* [22], *Zea mays* [23], and *Physcomitrella patens* [24, 25]. Members of both sub-groups of PLC have their own specific signature domains: PI-PLC group consists of X and Y catalytic domains, which leads to the formation of the TIM (triphosphate isomerase) barrel-like structure essential for the phosphoesterase activity, a calcium/lipid-binding domain (C2 domain) at the C terminal region, and EF-hand at N-terminal region to guide the binding of the enzyme to a membrane and PC-PLC contains only esterase domain.

*PI-PLC* genes regulate various cellular processes including signal transduction, cytoskeleton dynamic, vesicular trafficking, and remodeling of the cell by means of various lipid intermediates, the phosphatidic acid, diacylglycerol, inositol 1,4, 5-trisphosphate (IP3), and inositol hexakisphosphate (IP6) [11, 12, 26]. The members of the *PI-PLC* subgroups are activated by various stress conditions such as cold, salt, and drought stress [27–29]. The activation of *PI-PLC* during stress conditions has been reported in various plants such as *Zea mays*, where *ZmPLC1* gets upregulated and enhances grain production during dehydration and cold stress [23]. Similarly, *BnPLC2* of *Brassica napus* shows high expression in response to drought stress [18]. Genome-wide analysis of the *PLC* gene family in *B. napus* suggested that the overexpression of *BnaPI-PLC1* and *BnaPI-PLC2* as well as *BnaNPC1* genes enhances the DAG level under drought stress [19]. Knockout studies of *Arabidopsis thaliana*, *AtPLC3*, and *AtPLC9* result in enhanced sensitivity to heat revealing their role in thermotolerance [11, 30, 31]. Additionally, *PI-PLC*s of tomato were found to be involved in hypersensitive response (HR) and immunity exposure [14, 26]. The role in plant immunity is also established in *Arabidopsis thaliana* [32]. The *PI-PLC* is also found to play important role in gravitropism, plant hormonal responses, photosynthesis and flowering [18, 24]. In *Pyrus*, *PI-PLC* is involved in the maintenance of the self-incompatibility [33]. The *PC-PLC* (*NPC*) are responsible for lipid conversion during phosphate-limiting conditions [29]. These play essential role in number of physiological processes and various biotic and abiotic stress responses [34, 35]. In addition to this, it is found in rice that *PC-PLC*/*NPC* also affect the root architecture by brassinolide response [12].

During post-genomics era, the whole genome sequencing of *Phalaenopsis equestris *[36], *Dendrobium catenatum* [37] and *Apostasia shenzhenica* [38] plants leads to ample opportunities for genome-wide characterization of various gene families in these orchids. However, such studies are rare in case of orchids. *P. equestris* is a prized commercial plant due to its floral morphology. *D. catenatum* also has floricultural importance but it is mainly known for its antioxidant, immune modulation and vasodilation properties as reported in traditional Chinese medicines [39]. *A. shenzhenica*, a primitive terrestrial orchid, has evolutionary significance due to the presence of contrasting features to the general morphology of orchids, e.g., actinomorphic flowers, indistinct labellum, absence of pollinia, and resupination of ovary and rudimentary gynostemium, supporting its divergence from Orchidaceae [40]. Orchids, in general, are important plants for their floricultural and medicinal value. They are endangered of survival due to various environmental stresses and excessive exploitation for human use. The role of the PLC gene family in growth and development as well as in stress tolerance is well established. Several PLC members have been reported to be involved in various cellular processes and signaling networks, which are triggered by stressful environmental cues. This makes the PLC genes potential candidates for genetic engineering for the production of plants with enhanced growth and stress tolerance.

The present study on identification and characterization of the *PLC* gene family in orchids is proposed to provide a better understanding of the structure, function, and phylogenetic relationships of *PLC *genes which in turn can facilitate their functional characterization and utilization for the introduction of improved traits leading to better growth and stress tolerance in these immensely important plants.

## Methods

### Identification of *PLC* gene family proteins and analysis of primary structure

Phospholipase C protein sequences of *Arabidopsis thaliana* (AtPLC) and *Oryza sativa* (OsPLC) were used as query sequences and Blastp was carried out against the NCBI derived *P. equestris* [36], *D. catenatum* [37], and *A. shenzhenica* [38] protein database (https://www.ncbi.nlm.nih.gov/protein) [41]. The retrieved PePLC, DcPLC, and AsPLC sequences were then analyzed for the presence of X and Y catalytic domains, calcium/lipid-binding domain, and esterase domain with SMART server (http://smart.embl-heidelberg.de/ )[42]. The domain architecture was constructed using Expasy - Prosite (https://prosite.expasy.org/ )[43]. The conserved catalytic centers were located with the help of multiple sequence alignment using the MULTALIN tool (http://multalin.toulouse.inra.fr/multalin/ )[44]. The MEME suite server (http://meme-suite.org/tools/meme )[45], with preset parameters (maximum number of motifs — 05, number of repetitions — any, optimum motif width — ≥ 6 and ≤ 200) was used for the identification of conserved motifs.

### Determination of physical parameters

The physiochemical characterization of PLC protein sequences (molecular weight, aliphatic index, instability index, isoelectric point, and hydropathicity) were done using the Expasy-ProtParam server (https:// web.expasy.org/protparam) [46]. The sub-cellular localization of protein was predicted by CELLO v.2.5 (http://cello.life.nctu. edu.tw/) [47] and WoLF PSORT (https://www.genscript. com/wolf-psort.html )[48]. The signal peptide and transmembrane regions were detected using online server SignalP.4.0 (http://www.cbs.dtu.dk/services/signalp/ )[49] and TMHMM v.2.0 (http://www.cbs.dtu.dk/services/TMHMM/) [50].

### Phylogenetic analysis and ortholog prediction

The full-length PLC protein sequences (AtPLC, OsPLC, PePLC, DcPLC, and AsPLC ) were aligned with the MUSCLE program and the phylogenetic tree was then constructed using MEGA X tool (http://www.megasoftware.net/) [51] by the maximum-likelihood method at a bootstrap value of 1000 and the model selected was the Jones-Taylor-Thornton (JTT) model.

The orthologs for PePLC, DcPLC, and AsPLC protein sequences were predicted using local NCBI BLASTp search, each candidate PLC protein sequence querying independently against each other, and the best bidirectional blast hit with an *e* value less than 10^−5^ was selected [52]. Orthologs were also detected using OrthoVenn2 ( https://orthovenn2.bioinfotoolkits.net ) [53]^.^

### Gene structure and promoter analysis

The coding sequences (CDS), gene sequences, and promoter sequences were retrieved for each PLC protein from the NCBI database. CDS sequences and gene sequences were analyzed by using Gene Structure Display Server 2.0 (http://gsds.cbi.pku.edu.cn/ )[54] for the exon-intron architecture. The *cis*-regulatory elements of the PLC protein sequences were recognized in 1.5-kb upstream sequences using PLACE server (https://sogo.dna.affrc.go.jp/cgi-bin/sogo.cgi?lang=en&pj=640&action=page&page=newplace )[55]. Further analysis of promoter elements was carried out for the identification of common specific promoters using the Venn diagram tool GeneVenn (http://GeneVenn (sourceforge.net)/) [56].

### Duplication events prediction

The duplication events among *PePLC*, *DcPLC*, and *AsPLC* CDS sequences were predicted with the help of sequence similarity index obtained from the MUSCLE tool (https://www.ebi.ac.uk/Tools/msa/muscle/) [57]. The genes sharing ≥ 80% identity were considered duplicates [58].

### Expression analysis

The CDS sequences of *PLC* genes were used for the BLASTn search against the high-throughput RNA-seq data available at the SRA database (https://www.ncbi.nlm.nih.gov/sra) [59] for different tissues in *P. equestris* [leaf (SRX1074879), root (SRX1074875), stem (SRX1074876), flower bud (SRX1074880), sepal (SRX1806366), petal (SRX1806365), labellum (SRX1806348), pollinia (SRX2938663), and gynostemium (SRX1805894)]; *D. catenatum* [leaf (SRX2251517), root (SRX2938667), green root tip (SRX2251515), white part of root (SRX2251514), stem (SRX2251516), flower bud (SRX2251519), sepal (SRX2251513), lip (SRX2251518), pollinia (SRX2938662), and gynostemium (SRX2251512)]; and *A. shenzhenica* [tuber (SRX2938654), seed (SRX2938653), and pollen (SRX2938652)] [37, 38]*.* The total hits were counted and RPKM values (reads per kilobase per million) were calculated using the formula RPKM = (*C* × 10^9^)/(*N* × *L*), where *C* = number of reads mapped to the sequence, *N* = total mapped reads in the experiment, and *L* = exon length in base-pairs for the gene. Heat maps for the spatio-temporal expression of *PePLC*, *DcPLC*, and *AsPLC* genes were generated using Hierarchical Clustering Explorer 3.5 (http://www.cs.umd.edu/hcil/hce/) [60, 61].

### Molecular modeling

#### Secondary structures

To predict secondary structures (alpha helices, random coils, beta turns, and extended strands) of PLC protein sequences, the SOPMA secondary structure prediction tool was used (https://npsa-prabi.ibcp.fr/cgi-bin/npsa_automat.pl?page=/NPSA/npsa_sopma.html )[62].

#### 3D structure prediction

The three-dimensional structure of PLC protein sequences was predicted using homology modeling in the online Phyre2 server (http://www.sbg.bio.ic.ac.uk/phyre2/index.cgi) [63] and PyMOL (https://pymol.org/) [64] was used for the visualization of the protein 3D structure.

## Results

### Identification, ortholog prediction and domain analysis

**Fig. 1:**
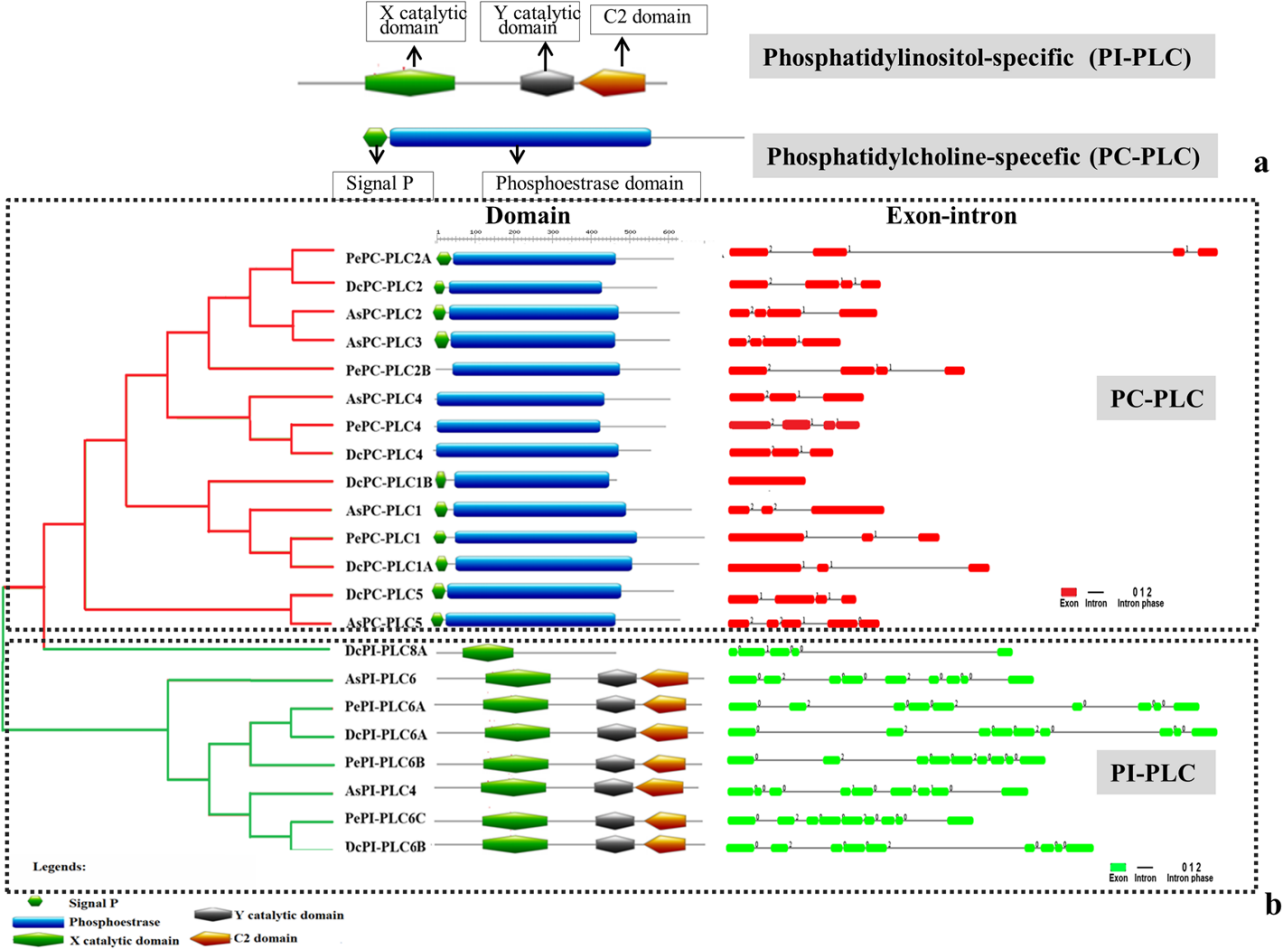
Domain and gene architecture analysis. **a** Specific domains of PI-PLC (X catalytic domain, Y catalytic domain, calcium/lipid-binding) and PC-PLC (signal P and phosphoesterase) categories. **b** Domains and exon-intron architecture for PI-PLC and PC-PLC for *P. equestris*, *D. catenatum*, and *A. shenzhenica*

**Fig. 2:**
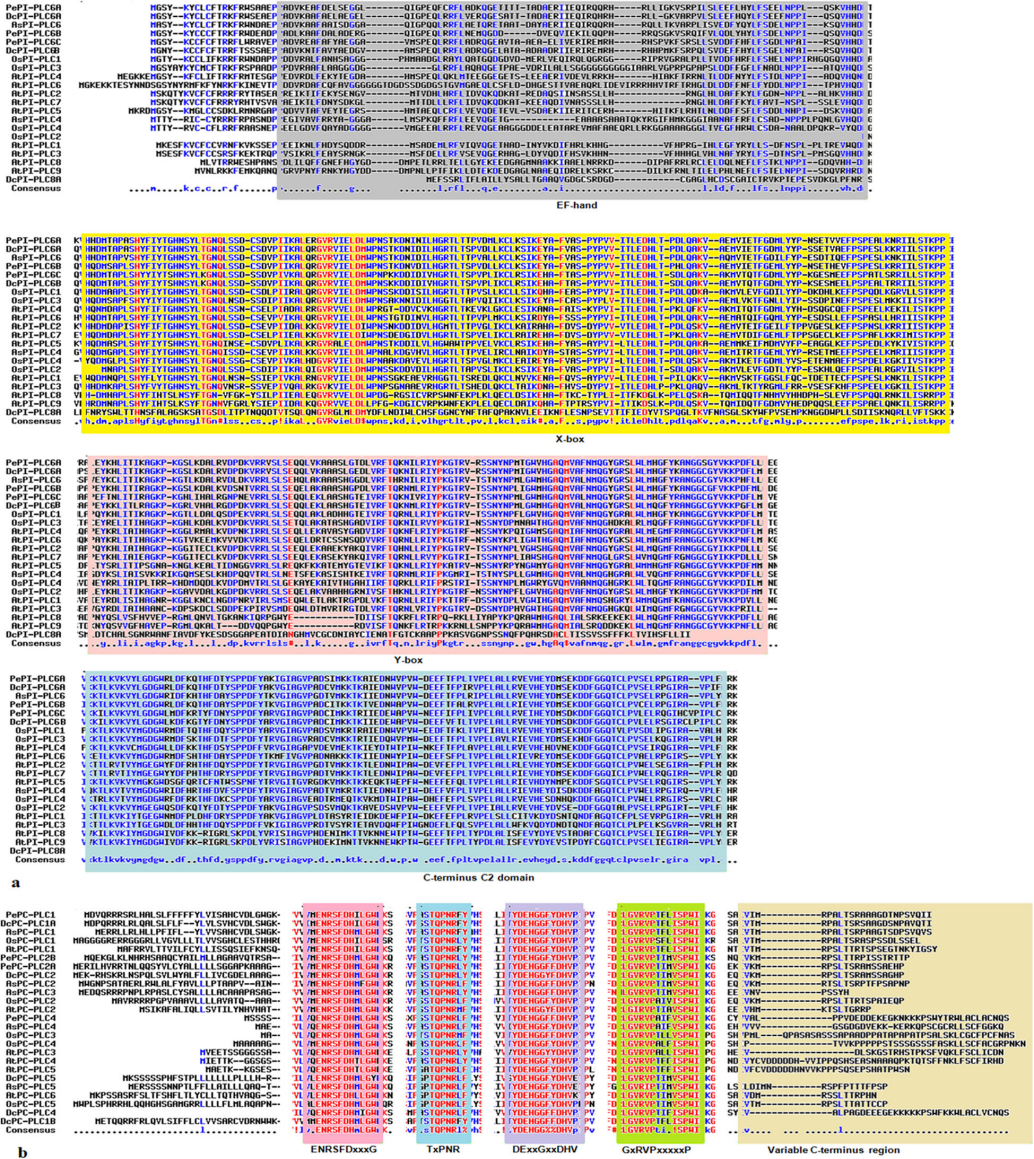
Multiple sequence alignment. Multiple sequence alignment of PLC protein sequences of *P. equestris*, *D. catenatum*, *A. shenzhenica*, *A. thaliana*, and *O. sativa* with the Multalin online tool. Highly conserved amino acid sequence regions are highlighted with boxes for both PI-PLC (**a**) and PC-PLC (**b**) sequences

**Fig. 3:**
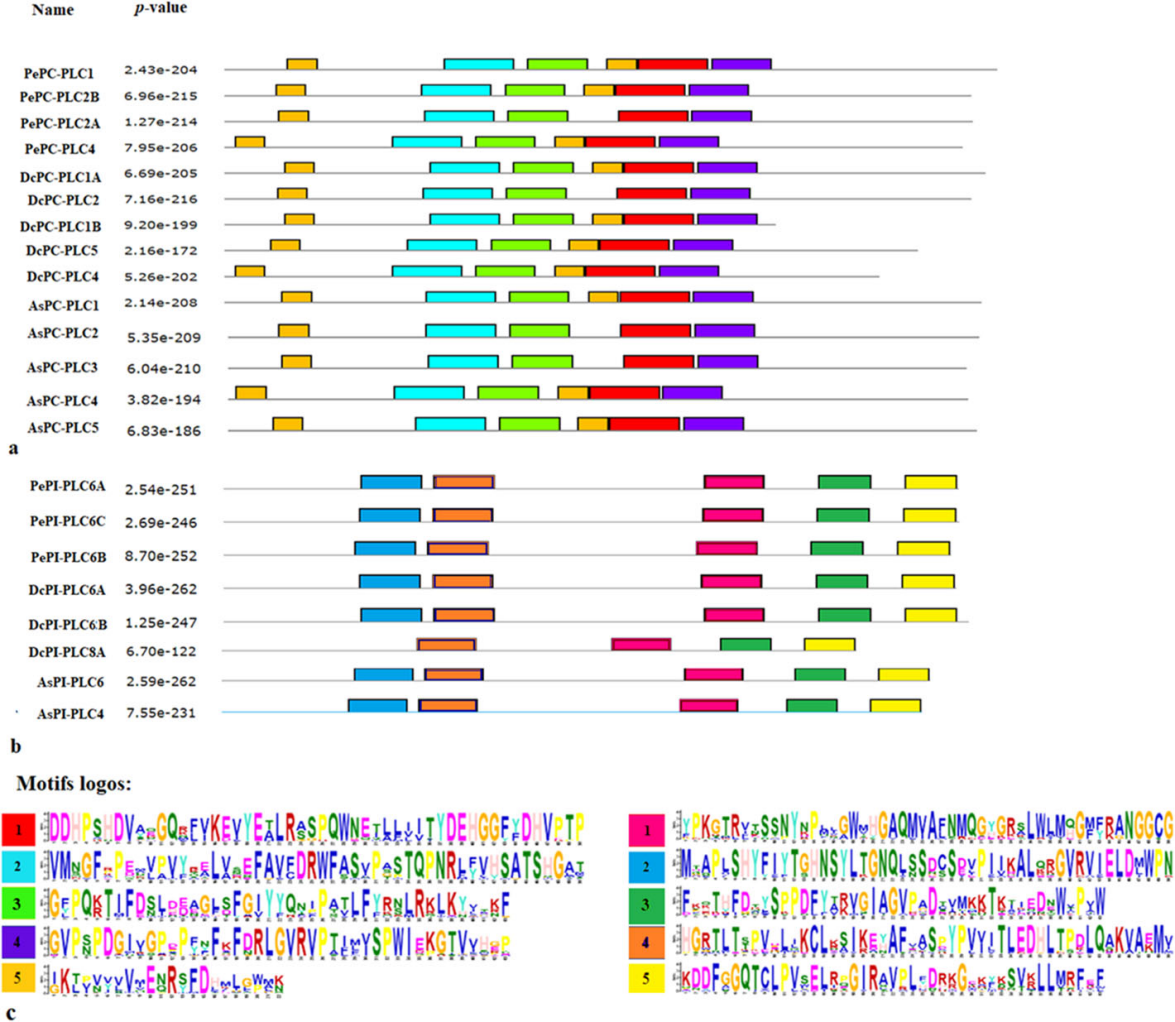
Motif analyses in PePLC, DcPLC, and AsPLC. Motifs were predicted by the MEME suite online server. **a** Conserved motifs in PePC-PLC, DcPC-PLC, and AsPC-PLC sequences, marked in colored boxes. **b** Conserved motifs in PePI-PLC, DcPI-PLC, and AsPI-PLC sequences, marked in colored boxes. **c** Sequence logo of PI-PLC and PC-PLC protein motifs obtained by the MEME server

Upon thorough exploration of *P. equestris*, *D. catenatum*, and *A. shenzhenica* genome, a total of seven, eight, and seven PLC sequences were predicted respectively. The *PLC* gene family in all three plants could be successfully divided into two major groups, phosphatidylinositol-specific PLC (PI-PLCs) and non-specific phospholipase C with specific catalytic activity for phosphatidylcholine (PC-PLC/NPC). The *PLC* gene family is represented by three *PI-PLC* and four *PC-PLC* genes in *P. equestris*, three *PI-PLC* and five *PC-PLC* in *D. catenatum*, and two *PI-PLC* and five *PC-PLC* in *A. shenzhenica*. Additionally, orthologous genes for *PePLC*, *DcPLC*, and *AsPLC* were predicted (Table 1). The nomenclature of proteins and their respective genes was done in accordance with their closest phylogenetic homologs in *A. thaliana* and *O. sativa* [10, 12]. Furthermore, structure analysis indicated that all the members of the PePI-PLC, DcPI-PLC, and AsPI-PLC groups comprised of X and Y catalytic domains and the calcium/lipid-binding domain. PC-PLC members were characterized by the presence of the phosphoesterase domain only (Fig. 1). Additionally, multiple sequence alignment showed the presence of a conserved region of EF-hand, X-box, Y-Box, and C-terminus C2 domain region in all PI-PLC sequences, whereas PC-PLC proteins were observed to have ENRSFDxxxG, TxPNR, DExxGxxDHV, GxRVPxxxxxP, and variable C-terminus region (Fig. 2). Motif analysis showed that all PLC protein sequences under study have the five highly conserved motifs. The conserved motifs are identified separately for both sub-groups of the PLC family (Fig. 3).

**Table 1:**
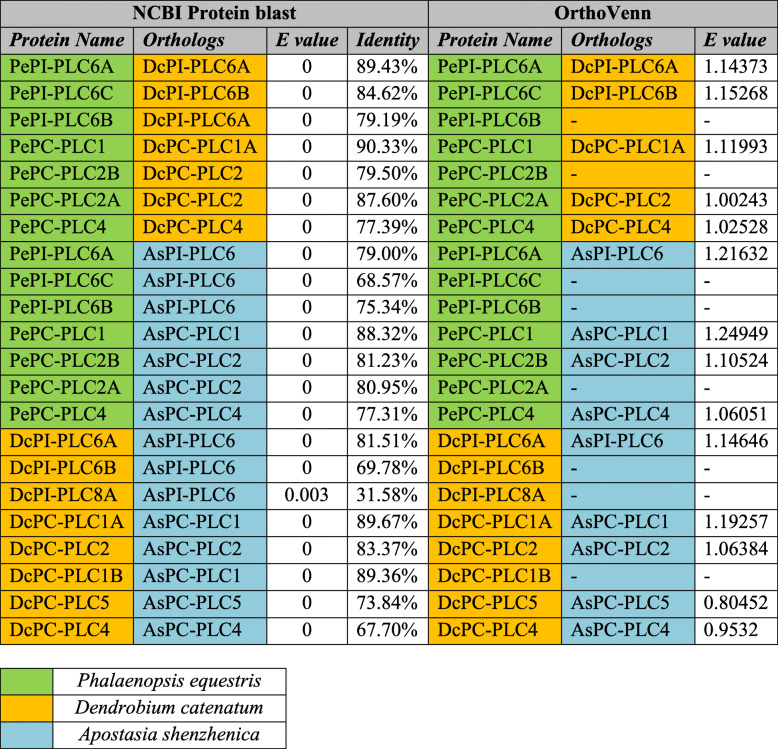
Ortholog prediction for PePLC, DcPLC, and AsPLC

In *P. equestris*, three protein sequences were identified along with isoforms with the help of the NCBI database search for protein gene ID. Every two proteins or a group of proteins sharing the same gene ID was considered an isoform. The PePI-PLC6C (XP_020579604.1; Gene ID: 110024153) was predicted to have two isoforms (XP_020579605.1 and XP_020579606.1), PePI-PLC6B (XP_020578229.1; Gene ID: 110023257) have one isoform (XP_020578230.1) and PePC-PLC2B (XP_020583114.1; Gene ID: 110026499) also have one isoform (XP_020583115.1); we have considered the longest isoform for further analysis, whereas no isoforms were identified for any DcPLC and AsPLC proteins.

### Protein characterization

Physico-chemical characterization of all PLC protein sequences was comparable in all three plants (Table 2). The average and range values for each physico-chemical property were calculated separately for both sub-groups: PI-PLC and PC-PLC. The peptide length for PePI-PLC ranged from 590 amino acid (aa) to 597aa, DcPI-PLC ranged from 594aa to 604aa and AsPI-PLC ranged from 588aa to 595aa with an average of 594aa, 599aa and 591aa, respectively. The DcPI-PLC8A has the smallest peptide length, which indicates its truncated nature, so this was not considered for average calculations. DcPI-PLC6B was predicted to have the longest protein sequence among all PI-PLC protein sequences in three orchid species. The average molecular weight for PePI-PLC, DcPI-PLC, and AsPI-PLC protein sequences was 67.8kDa, 68.1kDa, and 67.25kDa respectively. The isoelectric point for all the PI-PLC ranged from 5.04 to 6.46 with an average aliphatic index of 77 (Table 2). In the case of PC-PLC protein sequences, the average length for PePC-PLC, DcPC-PLC, and AsPC-PLC was 531aa, 481aa, and 524aa respectively. The molecular weight analysis showed that the average molecular weight of PePC-PLC, DcPC-PLC, and AsPC-PLC proteins was nearly equal to 59.2 kDa, 53.8 kDa, and 58.46 kDa, individually. The isoelectric point for all PC-PLC protein sequences ranged from 5.27 to 8.54. The average aliphatic index for protein sequences was 74.18. In addition to this, all the PLC proteins were observed to have a negative GRAVY value indicating their hydrophilic nature. Subcellular localization studies showed that most of the proteins were localized in the cytoplasm, nucleus, and mitochondria. Ten PC-PLC protein sequences were reported to have signal peptides (PePC-PLC1, PePC-PLC2A, DcPC-PLC1A, DcPC-PLC2, DcPC-PLC1B, DcPC-PLC5, AsPC-PLC1, AsPC-PLC2, AsPC-PLC3, and AsPC-PLC5). The transmembrane region prediction indicated the presence of a transmembrane region in four proteins (PePC-PLC1, DcPC-PLC1A, and AsPC-PLC2) (Table 2).

**Table 2:**
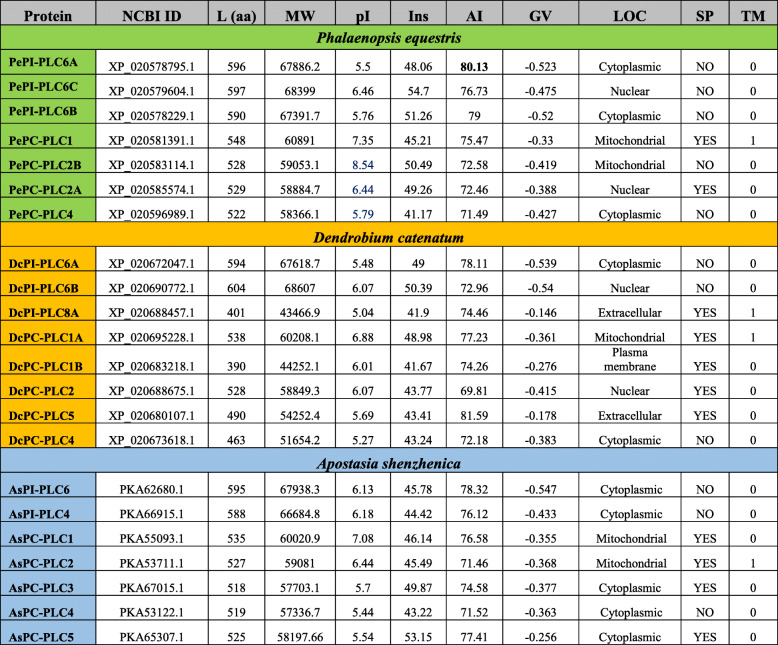
Physiocochemical characterization of PePLC, DcPLC, and AsPLC proteins

Isoelectric point (pI), protein molecular weight (MW) in kDa, instability index (Ins), aliphatic index (AI) grand average of hydropathy (GV), localization (LOC), signal peptide (SP) transmembrane domain (TM)

### Phylogenetic analysis

**Fig. 4:**
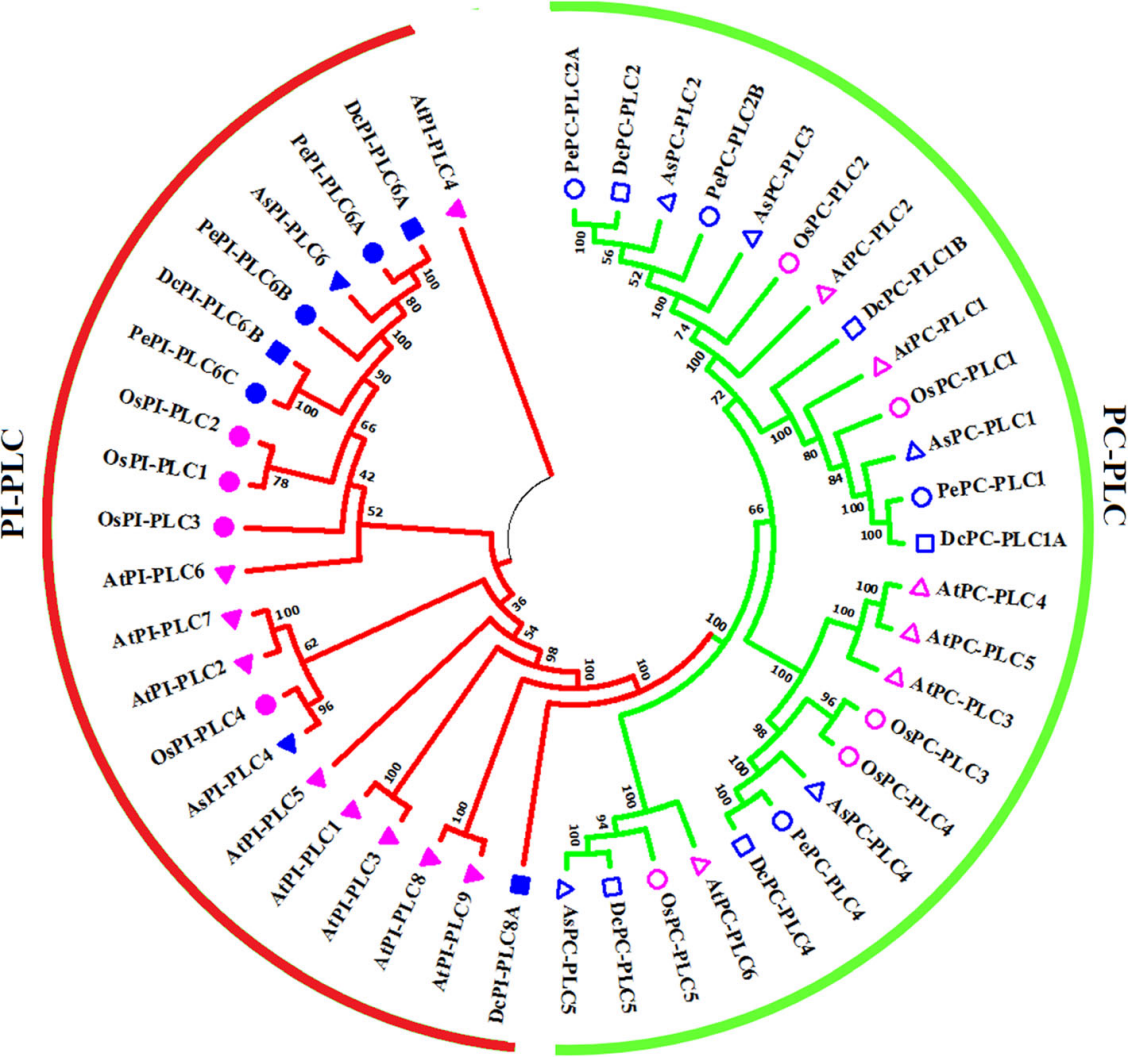
Phylogenetic analysis of PePLC, DcPLC and AsPLC proteins. Phylogenetic clustering of PePLC, DcPLC, and AsPLC protein sequences was done with AtPLC, and OsPLC sequences. The PI-PLC and PC-PLC groups are marked respectively in red and green

Phylogenetic analysis was performed for PLC protein sequences of *P. equestris*, *D. catenatum*, and *A. shenzhenica* by clustering along with protein sequences of *O. sativa* and *A. thaliana* to understand the evolutionary relatedness of this gene family to both dicots and monocots. All the proteins clustered along with their counterparts in the PI-PLC and PC-PLC sub-groups (Fig. 4).

### Genomic locus, gene structure, and gene duplication events

Genomic scaffold and stretch were identified for candidate genes and enlisted (Table 3). The exon-intron architecture analysis of *P. equestris*, *D. catenatum*, and *A. shenzhenica* showed the presence of nine exons and eight introns among all *PePI-PLC*, *DcPI-PLC*, and *AsPI-PLC* members. All *PI-PLC* genes of the three orchids were dominated by phase 0 introns indicating less disruption of the codon (Fig. 1), while in the case of PC-PLC, seven genes were predicted to have four exons and three introns, four genes with three exons and two introns, and one was with five exons and four introns. *DcPC-PLC1B* was intron-less (Fig. 1, Table 3). The genes of this PC-PLC sub-group were dominated by phase 2 introns, which indicates the disruption of the codon between the second and third bases. These studies also indicated that the maximum number of exons was asymmetrical in nature because they were flanked by more than one intronic phase (Fig. 1). There were no duplication events predicted in any of the plants (Table S1).

**Table 3:**
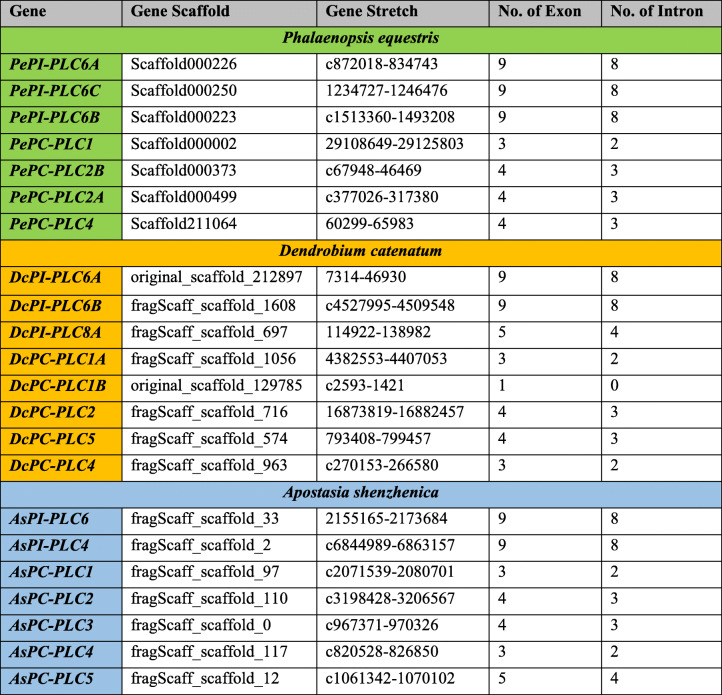
Gene characterization table of *PePLC*, *DcPLC*, and *AsPLC* genes

### Promoter analysis and spatio-temporal expression analysis

**Fig. 5:**
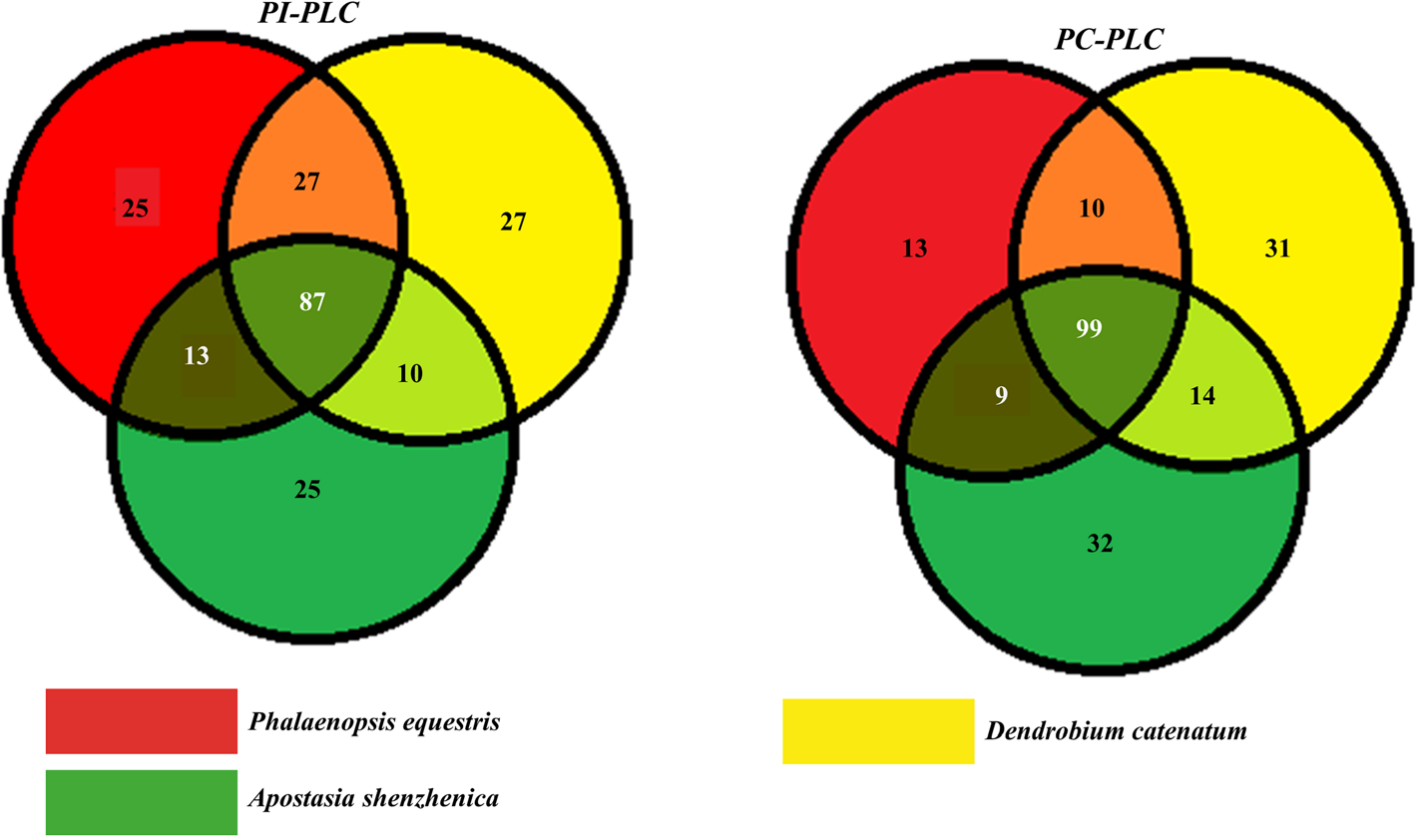
Promoter analysis: Venn diagram showing common numbers of promoters in *PI-PLC* (**a**) and *PC-PLC* (**b**)

**Fig. 6:**
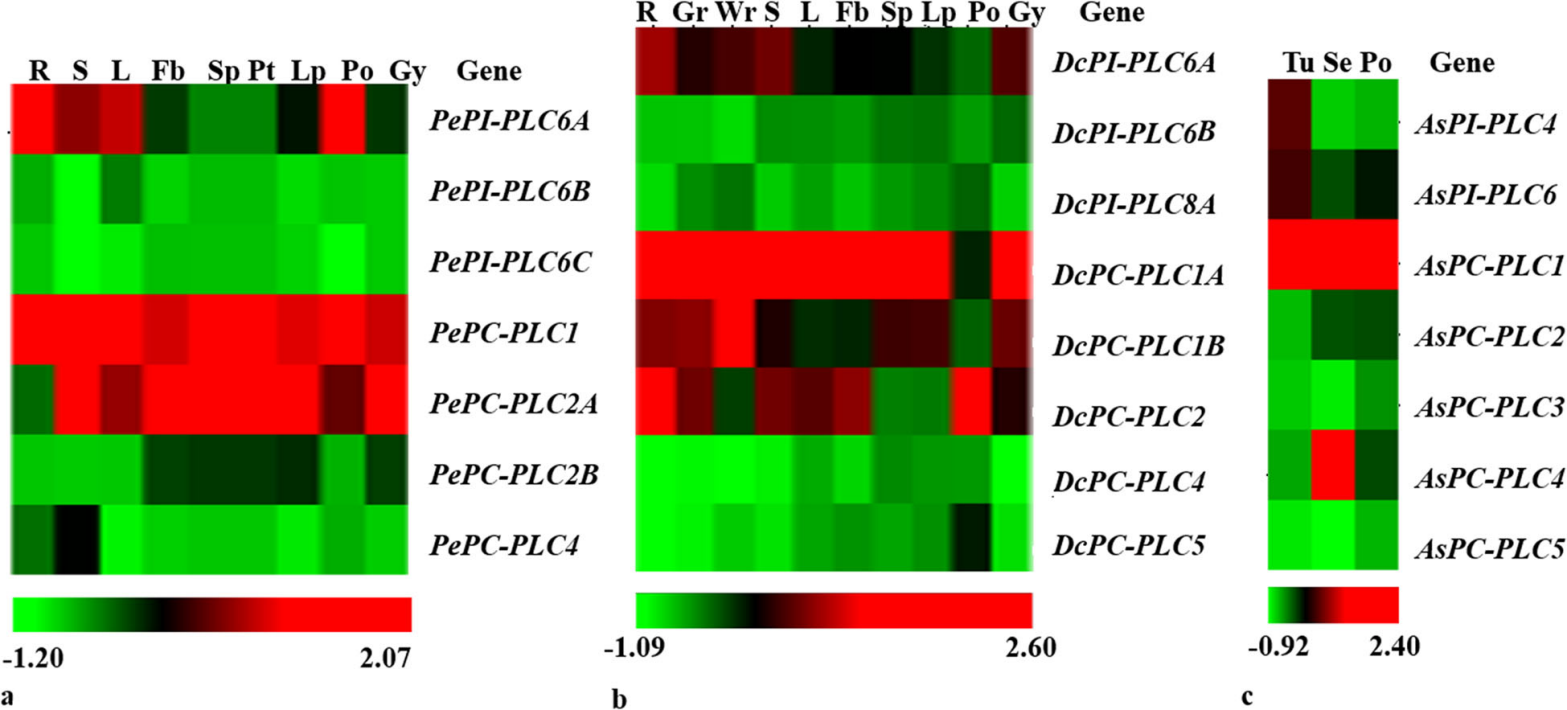
Expression profiling. The expression profile in the form of a heat map for (**a**) *PePLC* (**b**) *DcPLC*, and (**c**) *AsPLC* in different tissues such as leaf (L), root (R), green root tip (Gr), white part of the root (Wr), stem (S), flower bud (Fb), sepal (Sp), petal (Pt), labellum (Lp), pollinia (Po), and gynostemium (Gy), Tuber (Tu), and seed (Se) was generated by using HCE3.5 tool

The *PLC* gene sequences were analyzed up to 1500 bp upstream from the gene start site. The result showed the presence of conserved *cis*-regulatory elements within *P. equestris*, *D. catenatum*, and *A. shenzhenica* promoter region and this disclosed the significance of *PLC* genes in growth and developmental processes and stress responses (Fig. 5; Table S2). Detailed plant-wise analysis of promoter sequences of *Phospholipase C* genes revealed the presence of various *cis*-regulatory elements along with core promoter elements TATA-box (TATABOX5) and CAAT-box (CAATBOX1). The analysis showed the correlation function with the concerned genes were root-specific (ROOTMOTIFTAPOX1), mesophyll-specific (CACTFTPPCA1), pollen-specific (POLLEN1LELAT52), wound-activating W-box (WBOXNTERF3), WRKY proteins binding to W-box (WRKY71OS), dehydration-responsive (MYC), ABRE-like binding site motif (ABRE) involved in abscisic acid regulations, auxin-responsive elements (AuxREs), salicylic acid- and ethylene-mediating response elements (ASF1, ERELEE4) and cold-, drought-, and ABA-responsive elements (LTRE) with consensus core sequences ATATT, YACT, AGAAA, TGACY, CANNTG, ACGTG, TGACGTGGC/KGTCCCAT, TGACG, AWTTCAAA, and CCGAC. The presence of these elements accounts for the responsive nature of *PLC* genes under biotic and abiotic stresses and in various developmental processes (Table S2). The expression profile for *PePLC* and *DcPLC* was constructed on the basis of the RPKM value in various developmental stages like leaf, stem, root, sepal, petal, pollen, and gynostemium. Similarly, for *AsPLC* genes profile was constructed for tissues like tuber, pollen, and seeds. The analysis indicates that the *PePC-PLC1*, *DcPC-PLC1A*, and *AsPC-PLC1* genes had significant expression in all the tissues under study. *DcPC-PLC1A*, *DcPC-PLC2*, *PePC-PLC2A*, *and AsPC-PLC2* were showing outstanding expression in pollens, while *DcPC-PLC2* and *PePC-PLC2A* also have high expression in gynostemium. *AsPC-PLC4* had predominant expression in seeds, whereas expression in seeds is not studied in *P. equestris* and *D. catenatum*. The *PePI-PLC6A*, *DcPI-PLC6A*, and *AsPI-PLC6* were showing significant expression in vegetative tissues like leaf, root, stem, and tubers. The *PePI-PLC6A* and *DcPI-PLC6A* also had high expression in gynostemium, floral bud, and lip (Fig. 6).

### Molecular modeling

**Fig. 7:**
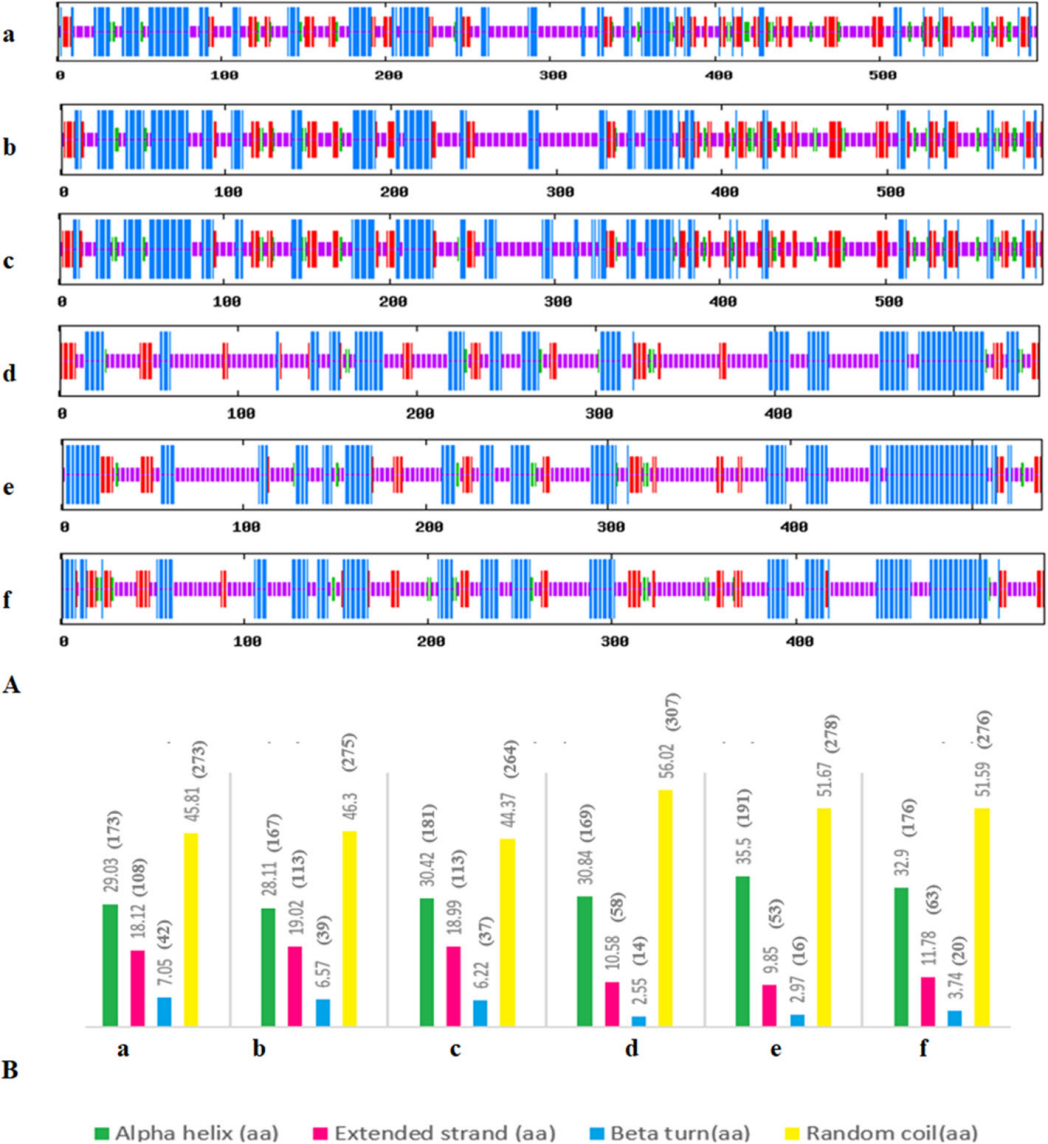
Structural analysis of proteins. **A** Diagrammatical representation of Secondary structures (a: PePI-PLC6A; b: DcPI-PLC6A; c: AsPI-PLC6; d: PePC-PLC1; e: DcPC-PLC1A; f: AsPC-PLC1). **B** Bar graph showing the percentage of alpha-helix, beta-sheet, and random coil of (a, b, c, d, e, f)

**Fig. 8:**
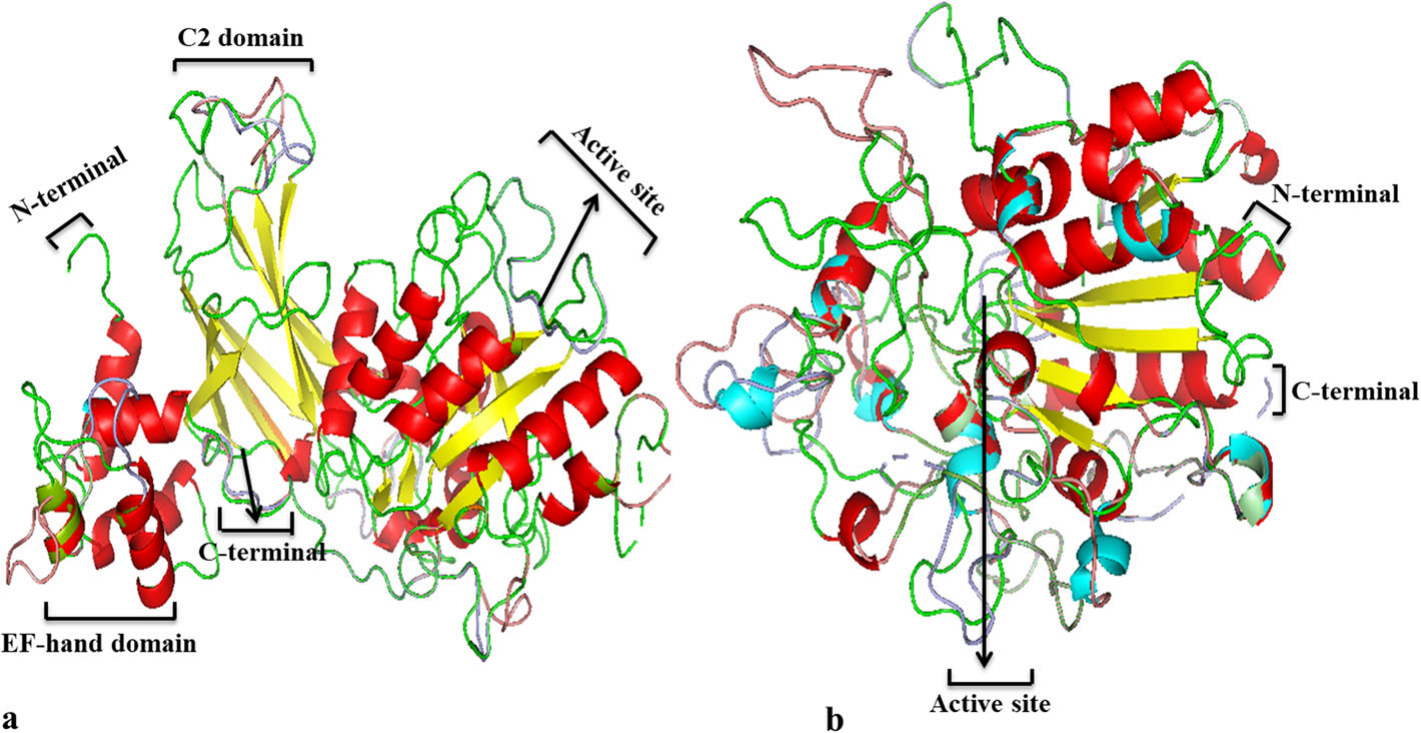
Simulated three-dimensional super-imposed structures. **a** Superimposed structure of PePI-PLC6A, DcPI-PLC6A and AsPI-PLC6. **b** Superimposed structure of PePC-PLC1, DcPC-PLC1A and AsPC-PLC1

Homology modeling is a technique, which provides new insights into protein structure and understanding the mechanism of protein function. A total of six proteins PePI-PLC6A, DcPI-PLC6A, AsPI-PLC6, PePC-PLC1, DcPC-PLC1A, and AsPC-PLC1 were taken for the comparative protein structure study from *P. equestris*, *D. catenatum*, and *A. shenzhenica* on the basis of their expression profile. The secondary structures were analyzed using the SOPMA server, which indicates that all the predicted PI-PLC and PC-PLC proteins were dominated by random coils ranging from 44.37 to 46.30% and 51.59 to 56.02%, respectively (Fig. 7). The random coils are often described as regions, where the folded chain acts more flexibly and dynamically than other secondary conformational structures. The secondary structure analysis indicated the proportion of alpha-helix, extended strand, beta-turn, and random coils in protein was almost the same in PePI-PLC and PePC-PLC proteins and their orthologs in *D. catenatum* and *A. shenzhenica*. The proteins taken from the PI-PLC group of *P. equestris*, *D. catenatum*, and *A. shenzhenica* were dominated by beta-sheets, each of them having 15 beta-sheets and 12 alpha-helix structures. The little variation at the EF-domain region in the sequence of AsPI-PLC6 and at N-terminal region in DcPI-PLC6A was observed. The members of the PC-PLC group were observed with six beta-sheet in their tertiary structure. But DcPC-PLC1A was predicted to have a large number of variations in their protein sequence at the alpha-helix region (Fig. 8). The superimposition of 3D-structure of PePI-PLC6A, DcPI-PLC6A, AsPI-PLC6, and PePC-PLC1, DcPC-PLC1A, and AsPC-PLC1 indicate their almost similar nature with little variation as indicated by the root mean square deviation (RMSD) value, which measures the average distance between the atoms of superimposed proteins (Table 4). RMSD values of the PI-PLC sub-group indicate that the variation in the structure of beta-sheets among PePI-PLC6A, DcPI-PLC6A, and AsPI-PLC6 protein sequences is comparatively less than alpha helices and random coils. However, in PC-PLC proteins, variations in the beta-sheets were observed to be more in comparison to the alpha-helix, except in PePC-PLC1 and AsPC-PLC1. This analysis showed that PLC proteins are conserved at structural level in *P. equestris*, *D. catenatum*, and *A. shenzhenica*.

**Table 4:**
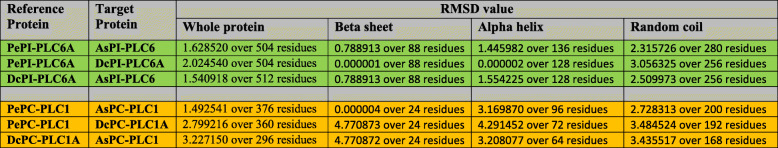
Root mean square deviation (RMSD) value of superimposed PI-PLC and PC-PLC proteins

## Discussion

The *PLC* gene family plays a major role in many critical cellular processes, including signal transduction, vesicular trafficking, cytoskeletal rearrangement, and secretion, which are necessary for plant growth and development, stress responses, and immune system maintenance [11, 12, 26]. Nevertheless, the reports on PLC transcription factors in orchids are not available till now. In the present study, *PLC* genes were identified from *P. equestris* [36], *D. catenatum* [37], and *A. shenzhenica* [38]. In our study of genome-wide exploration of *P. equestris*, *D. catenatum*, and *A. shenzhenica* through various bioinformatics techniques revealed the presence of seven, eight, and seven *PLC* encoding genes. The pattern of distribution of the number of genes among both sub-classes in the PLC family in orchids is somewhat similar to the rice [12]. However, there is a variation in the number of PLC genes in *Arabidopsis* (15), rice (9), and soybean (12) [5, 10, 12] (Table 5) and this difference in the size of the *PLC* gene family in monocots and dicots must be due to the loss of genes during the course of evolution. The whole *PLC* gene family is divided into two groups, PI-PLC and PC-PLC, on the basis of domain analysis, phylogenetic clustering, and homology modeling in accordance with reports on *Arabidopsis*, rice, tomato, cotton, and *Brassica napus* [9, 10, 12, 14, 19]. Like in *Arabidopsis* and rice, the PI-PLCs also have characteristic PI-PLC-X and Y catalytic domains and phospholipid-binding C2 domain at the C-terminal (Fig. 1). The conserved EF-hand region is observed in multiple sequence alignment of PI-PLC sequence of orchids, like rice and *Arabidopsis* [10, 12]. The identified PC-PLC proteins are predicted with a signature phosphoesterase domain, which consists of four conserved regions ENRSFDxxxG, TxPNR, DExxGxxDHV, GxRVPxxxxxP, and variable C-terminus region (Figs. 1 and 2). The physicochemical analysis of PI-PLC proteins of *P. equestris*, *D. catenatum*, and *A. shenzhenica* showed that the average length and weight of the PI-PLC proteins are in range with the PI-PLC proteins of rice [12]. Likewise, the average length and average weight of PC-PLC proteins fall within the range of those in *Arabidopsis* [10]. The signal peptides were predicted in 11 orchid PLC protein sequences, which is confirmatory with the presence of signal peptides in four proteins in *Gossypium hirsutum*, three in *G. arboretum*, and four in *G. raimondii* [9]. Furthermore, the evolutionary analysis of PePLC, DcPLC, and AsPLC along with PLC sequences of *A. thaliana* and *O. sativa* showed the clustering of PI-PLC and PC-PLC proteins in dedicated groups with high bootstrap values. The clustering of proteins with their closest relative indicated their conserved nature at the sequence level. The structural analysis for *PLC* genes and proteins showed the conserved nature of this gene family at the structural level as well. The exon-intron architecture revealed that all the PI-PLC members contain 7–9 exons. A similar kind of exon-intron pattern remains conserved among rice, *Gossypium* sp., *Brassica napus*, and *Arabidopsis*. However, in the case of PC-PLC, a number of exons ranged from 1 to 5, five genes had three exons, seven had four, one had five exons, and one had only one exon, which is also reported in PC-PLC members of rice, *Gossypium* sp., and *Brassica napus* [9, 12, 19]. Duplication event analysis indicated that the PLC genes of *P. equestris*, *D. catenatum* and *A. shenzhenica* did not participate in any significant duplication event. Similar, studies have been reported from rice where no duplication events were reported [12].

**Table 5:**
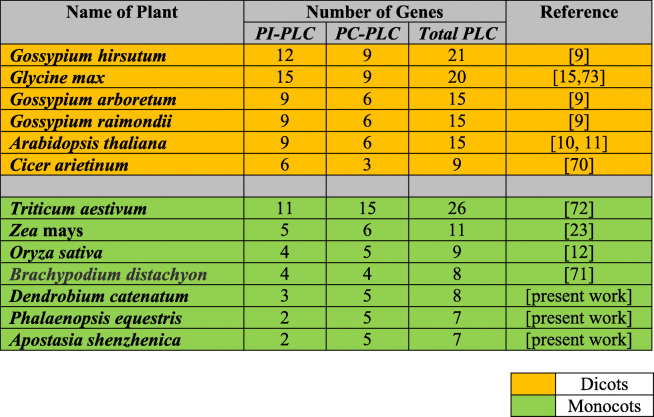
Size of the *Phospholipase C* (*PLC*) gene family in some dicot and monocot species

The promoter analysis of the *PLC* gene family in orchids indicates the presence of core promoter elements along with various other elements such as root-specific, mesophyll-specific, pollen-specific, stress-responsive, hormone-responsive elements and pathogen-responsive elements. These promoter elements are in line with functions performed by *PLC* genes. The dominance of abiotic stress (LTRE, PRE, MYC), hormone (ASF1, ERE), and pathogen (W-BOX, WRKY) responsive elements reflects the role of the *PLC* gene family in immunity responses and in both abiotic and biotic stress resistance. Similarly, the presence of *cis*-elements has been reported in the rice promoter with a predicted role in regulating gene expression patterns during abiotic stress conditions [12]. The presence of auxin-responsive *cis*-elements in the promoter region of the *PLC* gene family has also been observed in cotton [9]. Additionally, the presence of ASF-1and LTRE promoter elements, which are involved in the activation of various genes by auxin/salicylic acid-and ABA-mediated pathways and indicates the role of *PLC* genes in cold, drought, and salt stress responses [12, 65]. Similarly, the role of *PLC* genes (*OsPI-PLC1*, *OsPI-PLC3*, and *OsPI-PLC4*) is also reported in rice during cold, drought, and salt stress responses [12, 66]. The presence of W-box (TGAC) *cis*-elements in the promoter regions of *AsPLC*, *DcPLC*, and *PePLC* indicates the responsiveness of this family toward biotic stresses. The W-box has the capacity to bind with the *WRKY* transcription factor, which has a role in pathogen response [67] (Table S2). Expression analysis of *PLCs* indicated that these genes have differential expression in different tissues indicating their specific role in various physiological processes and developmental processes. In *Arabidopsis*, *NPC5* (*PC-PLC5*) expression is reported from the floral organ and the *PI-PLC2* gene is reported to have a role in reproductive development [68, 69], and in conformity, *AsPC-PLC5* showed moderate expression in pollen. The *PePI-PLC6A* and *DcPI-PLC6A* genes displayed significant expression in reproductive tissues (floral bud, pollen, lip, and gynostemium) in tune with the expression of their orthologs (*BnaPI-PLC6A3*, *BnaPI-PLC6A5*, *BnaPI-PLC6C3*, and *BnaPI-PLC6C4*) in *Brassica napus* [19]. In rice, *OsNPC4* showed higher expression in seed developmental stages while *OsPLC3* was downregulated in seed stages [12]. A similar interesting expression profile was observed in *AsPC-PLC4* and *AsPC-PLC3* as well (Fig. 6). The three-dimensional structure analysis in *Arabidopsis* indicates that the backbone of the tertiary structure of PC-PLC is made up of beta-sheets (which includes 7 beta structures) [6]; a similar trend is also observed in orchid PC-PLC proteins, which are composed of six beta-sheet structures surrounded by around 16 alpha-helix structures. The PI-PLC proteins of orchids are dominated by 15 beta-sheets and nearly 17 alpha-helix structures (Figs. 7 and 8).

## Conclusions

In this study, we have successfully done genome-wide characterization of the *PLC* gene family in three orchid species *P. equestris*, *D. catenatum* and *A. shenzhenica* through various *in silico* approaches. Total of 22 *PLC* genes were predicted in three orchid species, which were conserved at sequence and structure level. The expression profiles and *cis*-regulatory of all the *PLC* genes of three orchids were analyzed during various development stages. Both the expression analysis and promoter analysis indicate that the *PLC* gene family is involved in various developmental processes and stress responses. The study suggests that PLC is important for plant development and adaptation to various biotic and abiotic stresses.

## Supplementary Information

**Additional file 1: Table S1.**

**Additional file 2: Table S2.**

## Data Availability

Supplementary data associated with this article are available in the online version.

## Abbreviations

PLA: Phospholipase A
PLD: Phospholipase C
PLC: Phospholipase C
PI-PLC: phosphatidylinositol-specific phospholipase C
PC-PLC: Phosphatidylcholine-specific
pI: Isoelectric point
GRAVY: Grand average of Hydropathicity
MEGA: Molecular evolutionary genetics analysis
MEME: Multiple EM for Motif Elicitation
JTT: Jones-Taylor-Thornton
DAG: Diacylglycerol
PA: Phosphatidic acid
DGPP: Diacylglycerol pyrophosphate
IP6: Inositol hexakisphosphate
CDS: Coding sequences
